# An Imbalanced Fault Diagnosis Method Based on TFFO and CNN for Rotating Machinery

**DOI:** 10.3390/s22228749

**Published:** 2022-11-12

**Authors:** Long Zhang, Yangyuan Liu, Jianmin Zhou, Muxu Luo, Shengxin Pu, Xiaotong Yang

**Affiliations:** School of Mechatronics & Vehicle Engineering, East China Jiaotong University, Nanchang 330013, China

**Keywords:** imbalanced data, data expansion, continuous wavelet transform, synthetic minority oversampling technique, convolution neural network

## Abstract

Deep learning-based fault diagnosis usually requires a rich supply of data, but fault samples are scarce in practice, posing a considerable challenge for existing diagnosis approaches to achieve highly accurate fault detection in real applications. This paper proposes an imbalanced fault diagnosis of rotatory machinery that combines time-frequency feature oversampling (TFFO) with a convolutional neural network (CNN). First, the sliding segmentation sampling method is employed to primarily increase the number of fault samples in the form of one-dimensional signals. Immediately after, the signals are converted into two-dimensional time-frequency feature maps by continuous wavelet transform (CWT). Subsequently, the minority samples are expanded again using the synthetic minority oversampling technique (SMOTE) to realize TFFO. After such two-fold data expansion, a balanced data set is obtained and imported to an improved 2dCNN based on the LeNet-5 to implement fault diagnosis. In order to verify the proposed method, two experiments involving single and compound faults are conducted on locomotive wheel-set bearings and a gearbox, resulting in several datasets with different imbalanced degrees and various signal-to-noise ratios. The results demonstrate the advantages of the proposed method in terms of classification accuracy and stability as well as noise robustness in imbalanced fault diagnosis, and the fault classification accuracy is over 97%.

## 1. Introduction

Rotating machinery has been widely used as an indispensable part of industrial production [1]. The most noticeable factor in industrial production is safety [2], so the monitoring of the condition and diagnosis of malfunctions of rotating machinery have been a concern for more and more scholars [3,4]. The most common and easily damaged parts of rotating machinery are bearings and gears, which will lead to the paralysis of the entire mechanical system, property losses, and even casualties. Therefore, more advanced and universal fault diagnosis technology is urgently needed to identify faults in bearings and gears in rotating machinery so as to reduce losses [5,6].

To date, the most commonly applied methods for rotating machinery failure detection can be classified into three main groups: model-based [7], signal processing-based [8], and data-driven [9]. However, the model-based approach is challenging in establishing physical or mathematical models for relatively complex mechanical equipment [10]. Signal processing-based methods require a great deal of human knowledge to design some suitable features and understand the properties of the signals [11]. As such, these two techniques are difficult to promote in practical applications and have poor uniformity. On the contrary, the data-driven fault approach can effectively avoid the above disadvantages. It achieves bearing or gear failure classification and diagnosis by mining rules and connections within big data [12,13].

Deep learning, represented by convolutional neural networks (CNN), is a typical data-driven fault diagnosis method that enables end-to-end fault diagnosis without prior knowledge [14]. At present, researchers have applied CNNs in fault diagnosis of rotating machinery. For instance, Janssens et al. proposed a feature learning model for condition monitoring based on CNN [15]. Yao et al. used an acoustic approach and CNN based on a multiscale dialog learning structure and attention mechanisms for gear fault diagnosis [16]. Zhang et al. implemented bearing fault diagnosis under different operating loads using DCNN with original signals [17].

Although the work mentioned has obtained great diagnostic results, an issue remains to be addressed: CNN-based intelligent bearing fault diagnosis algorithms often require large samples for training. Nevertheless, obtaining enough fault samples in practical applications is difficult and even impossible, so the amount of data is usually imbalanced. This small and imbalanced data will considerably affect the accuracy of the fault diagnosis model.

In practical cases, rotating machinery has been in routine operation for a long time, and faults seldom happen during the machinery work. Consequently, faulty samples are more difficult to collect than normal samples, which results in the number of faulty samples will be much smaller than the number of normal samples [18]. Small and imbalanced data (S&I data) is a common situation faced by intelligent diagnosis models [19,20]. This situation is prone to cause model overfitting resulting in poor classification results, especially for deep learning fault diagnosis [21]. Thus, the diagnosis technique is more effective in classification when the amount of data is adequate, and the various types are balanced. For example, a mass of jobs conducted by other authors obtained promising results in the case of the Case Western Reserve University bearing dataset, which is typically a database of a sufficient and balanced number of samples [22,23,24]. Unfortunately, the scarcity of failure samples has permeated every aspect of our lives, such as in aerospace applications where rotating devices are replaced regularly, making it almost impossible to obtain failure samples, resulting in an extreme imbalance between the different categories. Therefore, sample augments and enhancement are the research focus.

The current mainstream sample expansion techniques, such as generative adversarial networks (GAN) [25], recurrent neural networks (RNN) [26], and variational auto-encoder (VAE) [27], have been widely applied. The above three mainstream methods have the potential to augment samples for specific problems. However, deep networks often require much time to train the model and are weak in generality. In addition, as a typical data expansion method, the synthetic minority oversampling technique (SMOTE) is able to solve the problem of data imbalance, which generates new samples between two adjacent samples by linear interpolation [28]. It compensates for the drawback that random oversampling inclines to cause overfitting. Han et al. adopted the Borderline-SMOTE method to oversample with a few class boundaries in the primary data [29]. Safe-Level-SMOTE multiplies the original few class instances by different weighting factors to construct safe regions [30]. The ADASYN algorithm adaptively adjusts the weights of different minority classes in the raw dataset [31]. The application of the above SMOTE algorithm directly performs sample generation on the original data. Nevertheless, the quality of the synthesized new samples largely rests with the original samples and their neighboring representatives. It is impossible to avoid suffering from the interference of noisy components and causing a shift in the data distribution, which will significantly affect the accuracy of the subsequent diagnosis.

Short-term Fourier transform (STFT) and continuous wavelet transform (CWT), as time-frequency analysis methods, can demonstrate the characteristic changes of the signal in the two-dimensional time-frequency spectrums and have better noise suppression [32,33]. As a result, the CWT and STFT are widely used for rotating machinery fault diagnosis. For example, Chikkerur et al. presented feature enhancement on fingerprint signals based on STFT [34]. Alexakos et al. achieved STFT denoising on motor-bearing image data [35]. Kankar et al. present a bearing fault diagnosis methodology using CWT, which consists of six different base wavelets [8]. However, the CWT is superior in extracting time-frequency features compared with STFT. The STFT adopts a fixed window function. When the window function is determined, its shape will not change, and the resolution of the STFT will be determined, resulting in its sampling interval cannot decrease with increasing frequency. In contrast, the wavelet transform has an adjustable time-frequency window [36], which can visually show the change in frequency components over time and accurately analyze the scale and resolution of periodic or transient signals. In addition, the CWT is the capability to detect weak defect signals from non-stationary data, even in strong noises [37,38]. Numerous researchers have adopted generative adversarial networks (GAN) to significantly expand the CWT-denoised image data to achieve better diagnostic results [39,40,41]. Nevertheless, GAN requires more cost to adjust the network structure to generate better samples and suffers from the problem that the model is not generalized. Compared with the SMOTE, the GAN algorithm requires more time to expand data and suffers from poor generalization.

Based on the above analysis, this paper chooses CWT as a tool for denoising and analyzing time-frequency features. In addition, the SMOTE was employed for sample expansion, thus proposing a new imbalance data augment the model with a time-frequency feature oversampling method (TFFO). Finally, CNN is established to realize the imbalance fault diagnosis of rotating machinery. The contributions of the research are listed as follows:

1. The proposed method performs a comprehensive data expansion from different dimensions. On the one hand, the sliding segmentation method partially expands some numbers of time-domain fault samples. On the other hand, SMOTE is applied to build a balanced dataset by expanding the minority fault samples in the time-frequency images.
2. CWT is employed as a pre-processing tool to construct 2-dimensional time-frequency images and denoise the data to enhance the stability of the features. In addition, an improved CNN based on LeNet-5 is established to extract the features and automatically recognize the fault location.
3. Compared with existing mainstream data augmentation techniques such as GAN and LSTM, the TFFO-CNN-based model has better performance in the diagnosis of bearing and gear failures under two imbalanced datasets, even under the interference of noisy environments.

The remainder of this paper is organized as follows: the introduction of SMOTE, CWT, and CNN in Section 2. Section 3 presents the general idea of the imbalance fault diagnosis model. In Section 4, two experimental studies are developed to evaluate the proposed approach for determining rotating machinery faults compared to other existing approaches. Finally, conclusions and future work are provided in Section 5.

## 2. Methodology

### 2.1. Data Expansion Based on Sliding Segmentation and SMOTE

#### 2.1.1. Sliding Segmentation

In actual practice, the machine is usually not allowed to run for long periods when a bearing or gear fails, resulting in a minimal number of vibrational fault signals that can be collected. Hence, finding a way to expand the limited signal is significant.

A sliding segmentation is employed for repeated sampling during the first data augmentation in this paper, which exploits the periodic nature of the fault signal to expand the sample. The process of selecting and moving the sliding window is as follows:

1. Window size. Theoretically, the size of the essential sliding window should be greater than or equal to one rotation period. Therefore, according to the rotation speed and the sampling frequency, the number of sample points produced by a rotation period of the bearing or gear can be calculated, that is, the minimum length of the sliding window.
2. Sliding step. The most basic principle for choosing the moving step size is that it should be less than one rotation period and that the step size should be smaller than the sliding window size. On the one hand, when the sliding step is small, the overlap rate of adjacent samples is higher, and the difference of expanded samples is slight, which is easy to cause overfitting of training. On the contrary, when the sliding step size is more extensive, due to the limitation of sample length, the expanded sample size is smaller, which is easy to cause training underfitting.
3. Starting point and sliding direction. In general, the first point of the raw data is set as the starting point of the sliding window on the premise that the data are correct. Until the last point of the data, the sliding direction should move in the direction of time.

**Figure 1:**
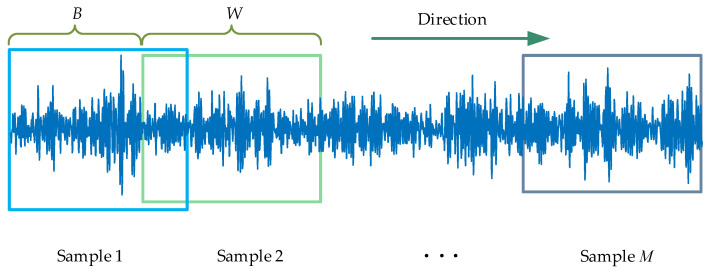
Illustration of the sliding segmentation. It mainly contains four key factors, including window size, sliding step and starting point, and sliding direction.

As depicted in Figure 1, Assuming that the sample length is *N*, the slip window size is *W*, the moving step size is *B*, and the number of samples after sliding segmentation is *M*, it can be expressed as:

(1) $$M=\frac{N-W+B}{B}$$

#### 2.1.2. Introduction to SMOTE

**Figure 2:**
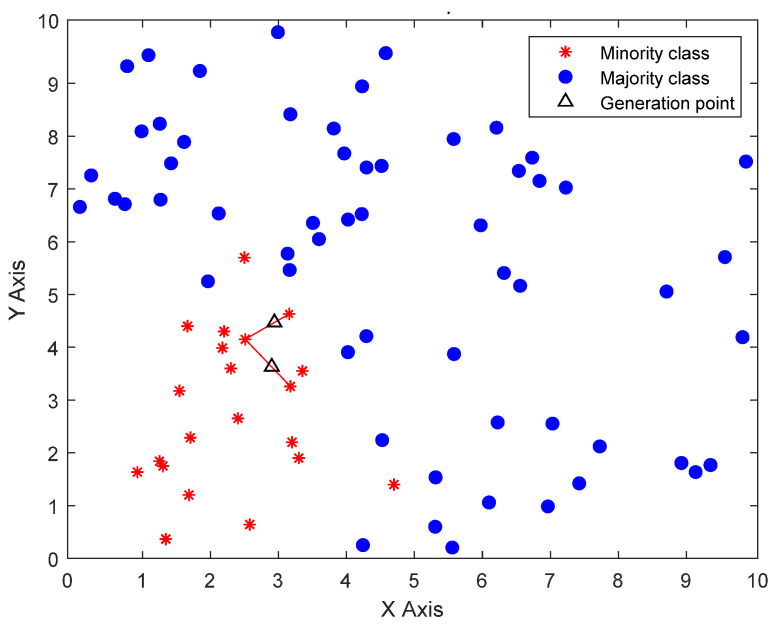
Illustration of SMOTE algorithm. The blue balls, red asterisks, and black triangles, respectively represent the majority classes, the minority classes, and the generation points.

The SMOTE is an improved scheme based on the random oversampling algorithm [28], as shown in Figure 2. The essential concept is to analyze the minority samples and add new samples to the data set. The approximate flow of the algorithm is based on the *K* nearest neighbor sample points of each sample point. It randomly selects *N* adjacent points to multiply the difference by a threshold in the range of (0,1) to achieve the purpose of synthesis of data. The process of the SMOTE algorithm is as follows:

1. For each minority category $X_{0}$, its distance from all surrounding samples is calculated on the basis of the Euclidean distance, and *K* nearest neighbor is obtained.
2. According to the sample imbalance ratio, the sampling ratio is set. For each minority sample, several samples are randomly selected from their *K* nearest neighbors.
3. For each randomly selected nearest-neighbor sample, create a new random point on the line segment connecting the pattern and the selected neighbor, as follows:
  (2) $$X_{new}=X_{0}+w(X-X_{0})$$
  where *w* is a uniform random variable in the range (0,1), $X_{new}$ is the generated point, $X_{0}$ is the minority category, X is the surrounding sample.

### 2.2. Introduction of CWT

#### 2.2.1. Wavelet Transform

Compared to the 1-dimensional time-domain signal, the 2-dimensional time-frequency domain matrix has more information as an image and can represent a more complex structure [42]. The one-dimensional time domain signal is converted into a two-dimensional characteristic spectrum by CWT in this paper. The CWT has excellent local description ability in the time and frequency domains [43]. Its temporal resolution and frequency resolution change with scale, which are in accordance with the characteristics of slow variations of the low-frequency signal and rapid variations of the high-frequency signal. CWT overcomes the shortcomings of the short-time Fourier transformation and continues its idea of time-frequency analysis of signals [44]. It is an excellent time-frequency analysis technique for transient analysis [45]. In fact, the bearing and gear fault signals contain many transient shock components [46]. Therefore, CWT has a unique advantage in dealing with rotating machinery failure datasets.

When the vibration signal:

(3) $$x(t)\in L^{2}(R)$$

Then the wavelet transform $w_{wt}(a,b)$ can be expressed as:

(4) $$w_{wt}(a,b)=\frac{1}{\sqrt{a}}\int_{-\infty }^{+\infty }x(t)\;\psi_{a,b}(\frac{t-b}{a})dt$$

where $\psi_{a,b}$ is a family of wavelet functions. It can be obtained from ψ(t).

(5) $$\psi_{a,b}(t)=\frac{1}{\sqrt{a}}\psi (\frac{t-b}{a})$$

where a is the translation factor, and b is the scale parameter. a,b∈R,a>0. In this paper, the size of b is set as the length of each sample.

#### 2.2.2. Selection of the Wavelet Basis Function

The selection of a wavelet basis function depends on the nature of the signal being analyzed and the purpose of the application. Among the existing wavelet functions, the Morlet wavelet has the form of an exponential attenuation vibration, which is very similar to the shock vibration response caused by bearing faults [47], so the Morlet wavelet has been widely studied in rolling bearing resonance demodulation technology. 

The Morlet wavelet basis function is composed of a complex trigonometric function multiplied by an exponential attenuation function, and the expression is as follows:

(6) $$\psi (t)=e^{-\frac{t^{2}}{2}}e^{j\omega_{0}t}$$

After stretching and translating, it can be expressed as:

(7) $$\psi_{a,b}(t)=\frac{1}{\sqrt{a}}e^{-\frac{{\left(t-b\right)}^{2}}{2a^{2}}}e^{j\omega_{0}\frac{\left(t-b\right)}{a}}$$

The acquisition of time-frequency images will be described in Section 4.

Following repeated sampling and expansion of some samples by sliding segmentation, the CWT is adopted to decompose the vibration signal of each sample into a wavelet coefficient matrix. The time-frequency distribution can characterize the joint information between the time and frequency domains and highlight the relationship between the signal and the operating state of the equipment. After the above processing, the signal benefits the model training and recognition.

### 2.3. Improved CNN Model Construction

**Figure 3:**
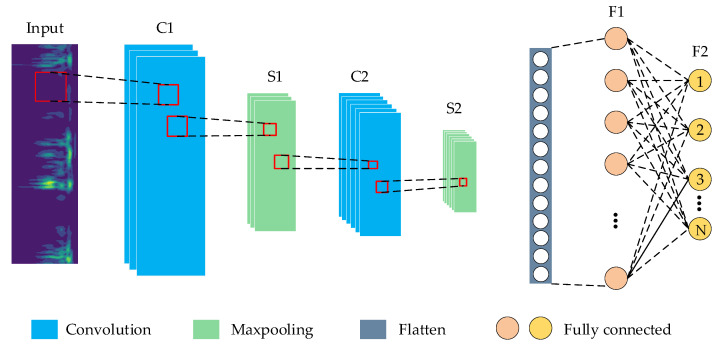
The architecture of LeNet-5-based CNN. It mainly contains two multiple convolutional, two pooling layers, and two fully connected layers. The time-frequency images are input to the first convolutional layer, and the classification of the output layer is achieved by the softmax function.

CNN has been developed rapidly in recent years and has become an efficient method for feature recognition [48]. CNN is composed of multiple convolutional, pooling, and fully connected layers, whose architecture is displayed in Figure 3. The structure of the CNN established in this paper is designed based on the LeNet-5 network [49]. The essence of CNN is to build a filter that can extract many different features of the input data. The output of the previous layer is used as the input of the next layer, and compelling feature extraction is achieved layer by layer.

**Figure 4:**
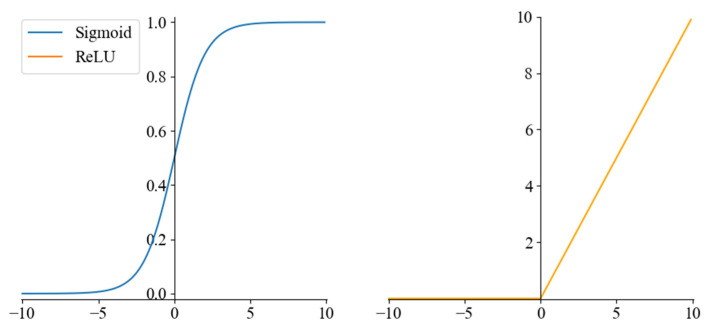
The Sigmoid and ReLU activation function.

In Figure 3, two convolution kernels of different sizes are constructed to extract the image’s main features and fine local features, respectively. The upper layer feature maps are convolved, and the Rectified Linear Unit (ReLU) activation function obtains the new feature maps. ReLU, as the most common nonlinear activation function in neural networks, can effectively improve the nonlinear fitting ability of neural networks [50], as shown in Equation (8). The Max-pooling layer uses the most significant local features to reduce the dimensionality of the feature input and compress the number of parameters after the convolution layer. The fully connected layer connects all features of the previous layer, integrates local information with the classification of the convolutional or pooling layer, and sends the output values after Sigmoid activation to the classifier. Sigmoid is a smooth and continuous activation function, also known as a logistic function, which can map a real number to the interval of (0,1) [51]. It is shown in Equation (9). The Sigmoid and ReLU activation functions are shown in Figure 4. Dropout is introduced to improve the model’s generalization ability and prevent overfitting [52]. The dropout algorithm randomly hides some units with a probability of failure during the training process [53]. Finally, the error loss between the predicted and actual values of the labels is calculated using a binary cross-entropy loss function for backpropagation, which has the ability to adjust the offsets in each layer to minimize the loss function.

(8) f(x)=max{0,x}

(9) $$g(x)=\frac{1}{1+e^{-x}}$$

Compared with the LeNet-5 network [49], the specific improvements of the improved CNN model in this paper are as follows:

(1) The LeNet-5 network uses a fixed 5 × 5 convolutional kernel, but the convolutional kernel is too large to extract the fine local features in the sample. In this paper, two convolution kernels of different sizes are constructed to extract the image’s main features and fine local features, respectively.
(2) To enhance the robustness of the model, the improved model adds a ReLU activation function after the convolution layer, which is useful to avoid gradient saturation and reduce the training time.
(3) The LeNet-5 network uses two fully connected layers, which is computationally intensive and time-consuming. Therefore, in the improved CNN in this paper, only one fully connected layer is used after the convolution module with the Softmax layer for output;
(4) A Dropout technique is added before the fully connected layer. This approach reduces the degree of correlation between neurons, thus avoiding network overfitting and improving the generalizability of the model.

## 3. Proposed Approach

**Figure 5:**
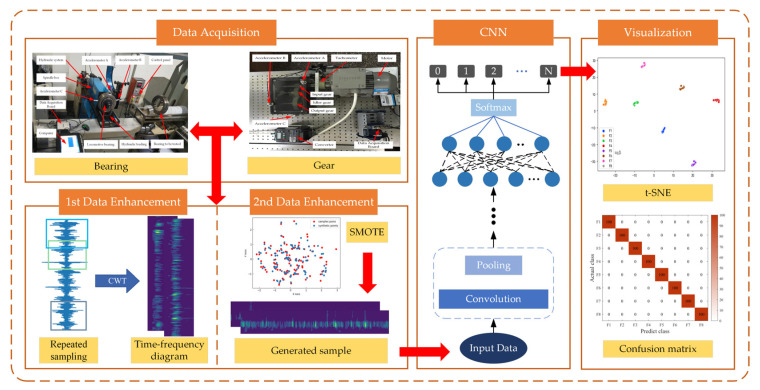
Imbalanced fault diagnosis flow chart of rotating machinery based on TFFO and CNN. First, the bearing and gearbox raw vibration signals are collected. Second, sliding segmentation is used for repeated sampling, and CWT is applied to generate time−frequency images. Third, the SMOTE is utilized to generate minority samples again. Finally, an improved CNN based on LeNet−5 is established to achieve intelligent fault diagnosis while the features are visualized by t−SNE, and results are displayed by a confusion matrix.

Aiming at the problem of reduced accuracy of model diagnosis due to S&I data, this paper proposes a new approach for imbalanced fault diagnosis of rotating machinery based on TFFO and CNN. Figure 5 shows the flowchart of the imbalanced fault diagnostic process, including the collection of acceleration signals and faulty signals expanded by sliding segmentation, the time-frequency feature extraction of the one-dimensional signals using CWT, the minority samples are balanced through SMOTE, illustration of CNN model, and visualization of the classification result. The main steps are described as follows:

1. Data acquisition. Bearings or gears experimental objects with different types of failure are loaded using different test benches. Acceleration sensors are installed to collect and construct vibration signal datasets.
2. First data expansion. On the basis of the above vibration signal dataset, slip segmentation sampling is performed to extend the range of samples. Moreover, CWT is applied to denoise and generate time-frequency maps containing rich information in time and frequency domains.
3. Second data augment. Samples from a few classes are analyzed to create new samples among the randomly selected nearest neighbor samples using SMOTE. The sampling rate is set according to the data imbalance rate to balance the time-frequency map dataset.
4. Diagnostic model. The time-frequency map dataset is fed into a designed CNN model comprising convolution, pooling, and fully connected layers with Softmax to output gear and bearing fault diagnosis results.
5. Visualization. The model output is visualized using the T-SNE algorithm and the confusion matrix.

## 4. Experiments and Results

In this section, experimental studies are conducted on bearing and gear, respectively: one is the locomotive bearing dataset, and the other is the public gearbox dataset from Zhejiang University. Meanwhile, the latest data expansion approaches are used for comparisons, such as GAN and LSTM. Moreover, the CNN model learning conditions and the diagnosis accuracy also deserve our attention. We apply t-SNE to project the features of each layer into a two-dimensional representation, which better describes the layer-by-layer learning capability of the CNN network model. The fault diagnosis results are quantified in detail by a multi-classification confusion matrix, and related charts will comprehensively demonstrate the fault recognition accuracy.

It is worth noting that this paper aims to simulate a realistic situation with a small number of fault samples, which provides a new idea for the imbalance fault real-time diagnosis of rotating machinery. Therefore, the model should use as few real fault samples as possible during the experiment. The author used only individual sensor data to construct the imbalance dataset in this paper’s bearing and gear fault diagnosis experiments.

### 4.1. Case Study 1: The Locomotive Bearing Dataset

#### 4.1.1. Experimental Setup

**Figure 6:**
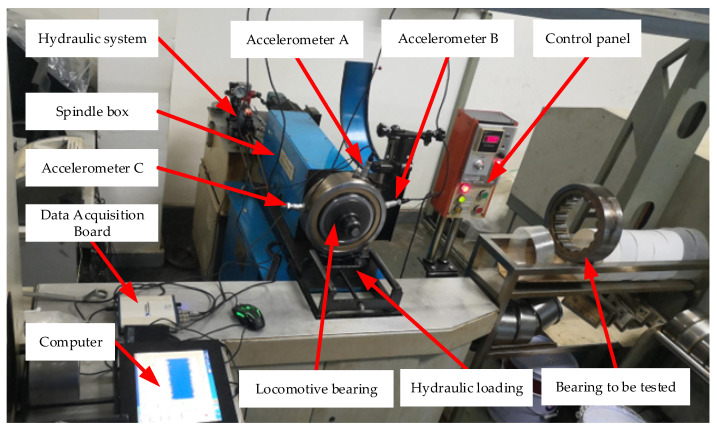
The locomotive bearing test rig. It is from a locomotive depot of the China Railway Administration. It mainly contains a hydraulic system, a spindle box, hydraulic loading, and three accelerometers at different locations.

**Figure 7:**
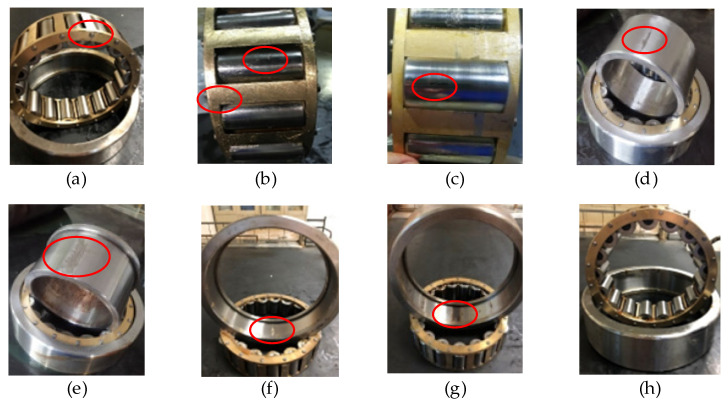
Different types of defective bearings: (**a**) F1; (**b**) F2; (**c**) F3; (**d**) F4; (**e**) F5; (**f**) F6; (**g**) F7; (**h**) F8. The red circle in the figure indicates the location of the defect.

The bearing data is employed from a locomotive depot of the China Railway Administration. The data set of bearing faults are real faults, not artificial processing faults. The current locomotive bearing dynamic detection system model of the Railway Bureau is the JL-501 series. The main body of the bearing detection system consists of the bearing test rig and the software detection device, as shown in Figure 6. The locomotive wheelset bearing is driven and loaded with the detection platform in this paper. The spindle speed is set at 500 rpm, and the radial load is 1.4 MPa. The locomotive bearings used in the experiment are NJ2232WB series cylindrical roller bearings with an outer diameter of 290 mm and an inner diameter of 160 mm. Vibration signals are obtained by three model CA-YD-187T accelerometers fixed at the outer ring of the bearings and a Ni-USB-4431 acquisition card. The sampling frequency is 20 kHz. Eight types of locomotive bearing failures, including normal state, are shown in Table 1, and the corresponding locomotive bearings are shown in Figure 7.

#### 4.1.2. Preprocessing of Data and Parameter Selection

**Figure 8:**
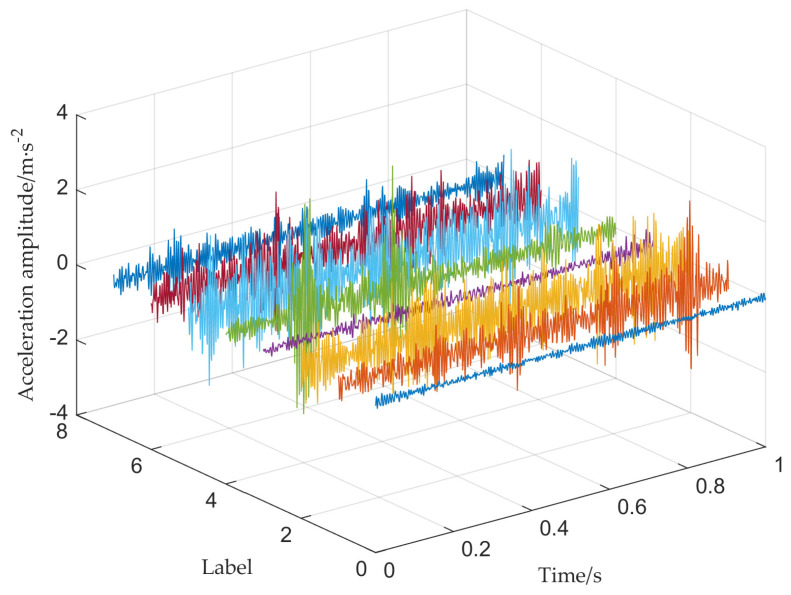
Time domain signal of F1−F8. It mainly contains eight types of fault signals in Table 1.

For the bearing data set of 8 categories, Figure 8 shows the corresponding time-domain signals. There are 1,200,000 data points for healthy bearings and 102,400 for the other seven types of fault data. According to the sampling frequency of 20 kHz and the speed of 500 rpm, the sample length of this experiment is 2400. Thus, this bearing data set has about 42 faulty samples and about 500 normal samples.

Three different imbalanced levels datasets are constructed artificially based on the number of normal bearing samples, where Dataset 1 has 50 normal samples, Dataset 2 has 250, and Dataset 3 has 500. The imbalance ratios for the normal and faulty samples of the three datasets are 1, 0.2, and 0.1, respectively. The specific process of building the three datasets is as follows:

In addition to the normal bearing samples F8, the remaining seven types of fault samples were expanded to build a balanced data set. In the first data expansion using the sliding segmentation method, the window size was 2400, and the moving step size was 2000. The number of repetition points was 400. Finally, the original samples were expanded to form Dataset 1. In the second data augment using the TFFO, the sample size was increased by different multiples for the Dataset 2 and Dataset 3 of the different imbalance ratios in Table 1. Ultimately, the number of samples for each category remained consistent with the number of samples for the bearings in the healthy state.

**Figure 9:**
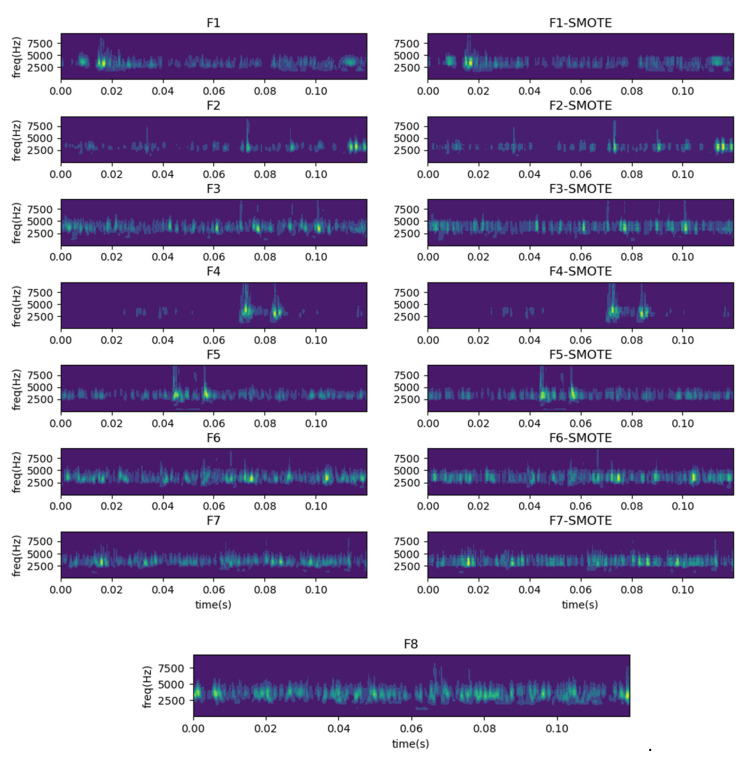
Time-frequency images of the original samples and generated samples. It mainly contains a healthy-bearing sample and seven fault-bearing samples, and seven generated samples.

One-dimensional time-domain signals are transformed into time-frequency feature images using CWT, where the scale factor is set to 2400, depending on the length of each sample. The frequency range of the vertical axis in the time-frequency diagram indicates the fault resonance frequency range (2.5 kHz–5 kHz), which is determined by the fault itself. For example, the resonant frequency of the bearing refers to the fact that the bearing rotation will cause a shock at the fault location, and this shock will produce the phenomenon of inherent frequency resonance. Figure 9 shows the time-frequency images of the original and generated samples after the transformation by CWT. The differentiation between the various types of samples is still evident in Figure 9. We can see that the fault feature information is mainly distributed in the middle frequency band (2.5 kHz–5 kHz), and the generated time-frequency image is similar to the primitive image under the same health state.

There is no need to make the generated time-frequency sample utterly consistent with the original ones. The identical samples are meaningless in the training process of the model. Fortunately, the vibration signals of bearings and gears are distinctly periodic. Thus, the model can perform highly accurate fault diagnosis when the generated samples contain comprehensive fault information. In addition, many studies took flip, rotate, and randomly crop as image data augment tools to make different samples [53,54,55].

TFFO is a method of oversampling based on feature space, in which a new sample is formed by synthesizing new characteristics between a primitive sample and the nearest neighbor. The distribution of the data generated by TFFO technology is very similar to the original data, which causes the generated and the original picture to be challenging to distinguish and recognize by human eyes. However, this is not difficult for CNN.

After secondary data expansions, the class-balanced dataset was divided into three parts: 60% for training, 20% for validation, and 20% for testing. What needs to be emphasized is that the test set data is fixed and does not contain any generated samples, while the training and validation sets are randomly assigned from the remaining samples in proportion to the remaining samples. Subsequently, the data are input into 2-dimensional CNN for fault location identification. In order tto reduce the effect oerrors; e errors, ten random experiments are passed to maximize accuracy and minimize loss of validation set data. The trained model is then employed to classify the data from the test set. The choice of hyperparameters in the CNN model significantly influences the accuracy of subsequent fault diagnosis. In this paper, the epochs, batch size, learning rate, and dropout were 60, 50, 0.001, and 0.5, respectively. The structure and parameters of CNN are described in Table 2.

The software and hardware facilities used for data processing in this experiment are as follows: Win10 64-bit operating system, AMD Ryzen 7 3800X 8-Core processor, 32 GB running memory, a program running Python3.6, Spyder, Tensorflow1.13.1.

#### 4.1.3. Diagnosis Results and Visualization

**Figure 10:**
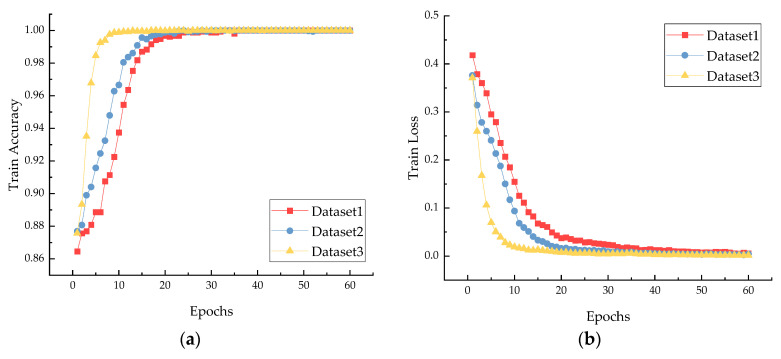

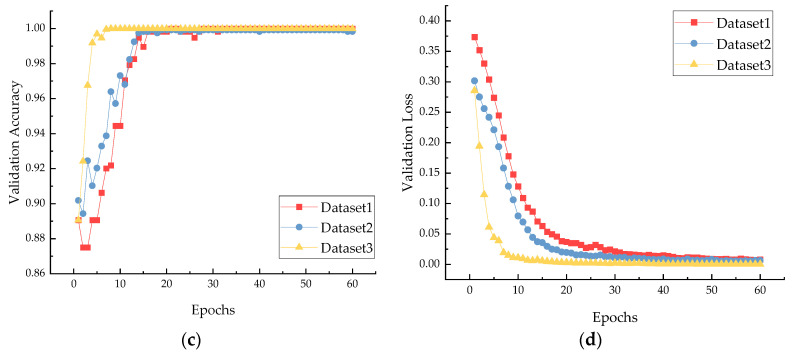
Experimental results for balanced bearing Dataset 1, Dataset 2, and Dataset 3: (**a**) train accuracy; (**b**) train loss; (**c**) validation accuracy; (**d**) validation loss.

Figure 10 shows the loss and accuracy curves after balancing Dataset 1, Dataset 2, and Dataset 3 using the proposed TFFO and CNN methods. In all datasets, the loss value decreases to about 0.01, and the accuracy rate reaches 100% when the iteration reaches the 40th round. From the 40th round onward, the model further converges until it is stable. We can clearly learn that the model has promising diagnostic results and strong generalization performance.

**Figure 11:**
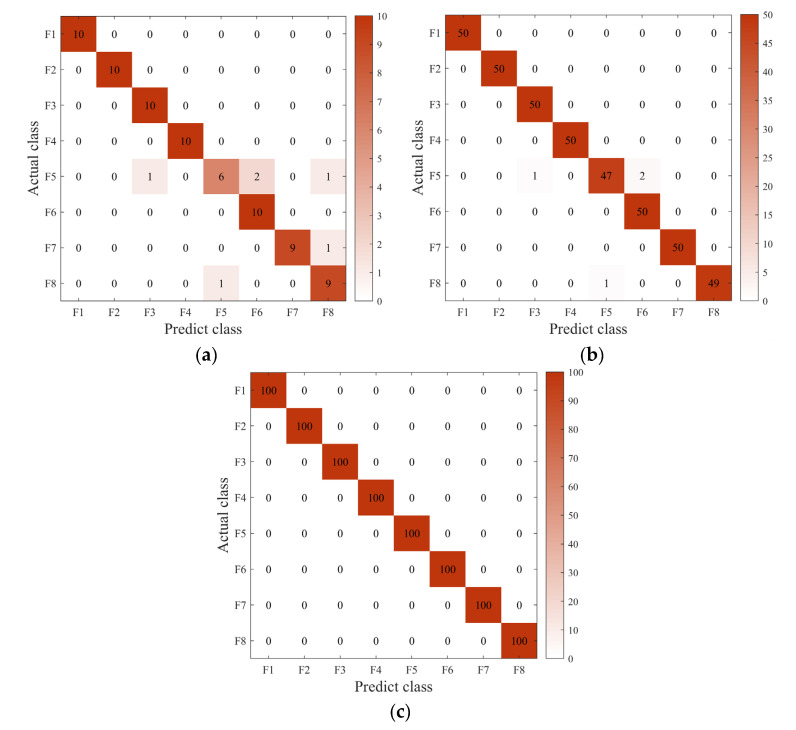
The confusion matrix under different datasets: (**a**) Dataset 1; (**b**) Dataset 2; (**c**) Dataset 3.

A multiclassification confusion matrix is introduced to conduct a detailed quantitative analysis of fault diagnosis results, which provides a comprehensive view of the types and the specific number of misclassifications of the actual fault types. Figure 11 visually represents the classification of the test set after sample balancing for the three data sets in Table 1. Figure 11a shows the classification results for the test set in Dataset 1. There are ten samples for each fault type, and the categories F5, F7, and F8 are misclassified with a misclassification rate of 7.5%. The imbalance ratio of Dataset 2 is 5 to 1 in Figure 11b. After the dataset is balanced, the sample size increases significantly, and the misclassification phenomenon is much improved than in Dataset 1. From Figure 11c, it can be observed that the result is satisfactory under Dataset 3. The final accuracy reaches 100%, although the Dataset 3 sample ratio reaches 10 to 1, and the imbalance is very high.

Figure 10 and Figure 11 explain more intuitively the effect of class imbalance on the final classification accuracy, which shows that the balance between different data types significantly affects the final accuracy. The loss curve shows that the model converges faster after secondary data expansion. The accuracy curves and confusion matrix results show that the model is more stable and more accurate after data balancing.

T-SNE (T-distributed stochastic neighbor embedding) algorithm is a nonlinear manifold learning algorithm to visualize high-dimensional data [56]. The algorithm aims to keep the neighborhood distribution characteristics of high-dimensional data and low-dimensional data consistent as much as possible. The KL divergence is used to measure the difference between two distributions, and the gradient descent method is used to minimize the distribution difference.

**Figure 12:**
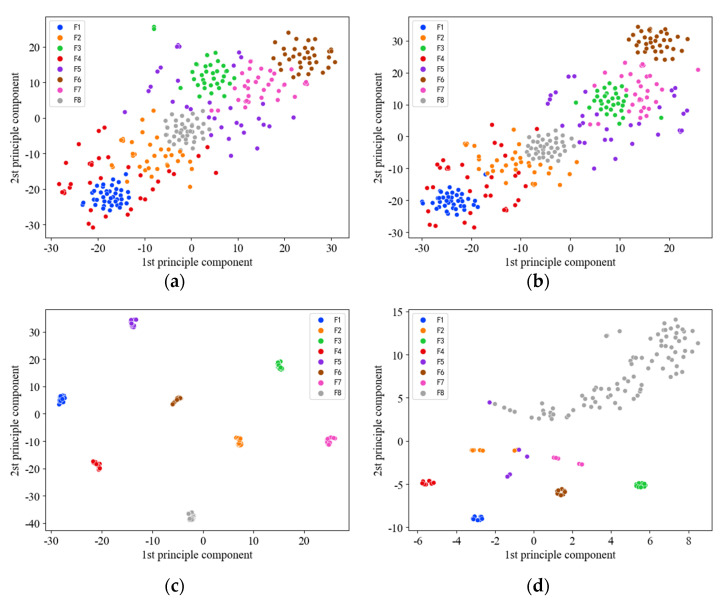
The visualization by t−SNE of the learned features in the Conv2D layer and Fully connected layer of Dataset 3: (**a**) layer C1 in the balanced Dataset 3; (**b**) layer C2 in the balanced Dataset 3; (**c**) layer F1 in the balanced Dataset 3; (**d**) layer F1 in the imbalanced Dataset 3.

T-SNE dimension reduction was performed on two convolution layers and a fully connected layer to visualize the model effect in 2dCNN. As can be seen from Figure 12a,b, the distribution among the eight classes of samples is disordered and covers significantly. It is impossible to distinguish the types of faults. However, the situation gradually improves as the number of layers in the network increases.

Figure 12c shows the sample distribution of the last fully connected layer. There is a clear distinction between different types of faults and no misclassification. Nevertheless, the original imbalanced data input with the same parameters and network structure of 2d-CNN, its full-connection layer classification effect is still not ideal. The label F8 in Figure 12d is the normal sample. It is impossible to minimize the intraclass distance due to the large proportion of imbalances leading to a more dispersed distribution. Several samples labeled F5 were mistakenly assigned to other areas, leaving some scattered and accessible.

In addition, we constructed a series of experiments to compare and analyze the proposed model. First, we show the average accuracy of the proposed approach ten times under different imbalance ratios and noise levels.

Gaussian white noise is added to the original signal to generate noisy signals with different signal-to-noise ratios (SNRs) to simulate the industrial environment. Then different imbalance ratio data sets are constructed and inputted into the proposed model for data augment. All experiments were expanded on the original scaled data using TFFO until the categories were balanced. Each group of experiments calculated the average accuracy of 10 tests and the extreme range between the maximum and minimum accuracy. The average accuracy rate reflects the accuracy of the model. When the value is more significant, the model is more accurate. Moreover, the extreme range indicates the degree of generalization of the model, and the smaller the value, the better the generalization effect. It is worth noting that the amount of bearing data in the healthy state is much more considerable than in the faulty state. Hence, the imbalance ratio can reach 10 to 1.

Table 3 shows the test accuracy of the proposed method in different SNRs. We can learn that after using the proposed TFFO for data augment, the performance of 2dCNN in classifying imbalanced data has been significantly enhanced, and the test accuracy of Dataset 2 and Dataset 3 reaches 97% and 99% or more, respectively. On the contrary, the test accuracy of Dataset 1 is between 91% and 98%, which is not a satisfactory result. The number of expanded samples increases as the imbalance ratio continues to increase. Subsequently, the average accuracy at an arbitrary SNR is increasing. A satisfactory result of 100% accuracy was achieved using Dataset 3 in a 0 dB noise environment.

Through the analysis of the experimental results, it is easy to find that TFFO and 2dCNN can overcome the data imbalance problem well. On the other hand, we show the 10-fold average diagnostic accuracy of different methods at different noise levels using Dataset 3. In this section, in order to validate the proposed imbalance fault diagnosis model, the proposed method was compared with two mainstream data enhancement algorithms: GAN [57] and LSTM [58]. The two prevalent networks are broadly described as follows:

The generators and discriminators in the GAN have constantly been adversarial and improved [59,60]. Random input noise is eventually converted into a signal similar to the target output. Different classes of faulty samples are inputted into the GAN until the number of faulty samples equals that of normal samples.

LSTM is an improved network based on recurrent neural networks. It can predict the next data point based on the correlation of the temporal signal. The process is repeated until a fault signal with the same length as the normal signal is generated. In this paper, the structure of LSTM is 1000-32-32-1, the Dropout is 0.2, and the batch size is 16. Adam is selected as the optimizer.

**Figure 13:**
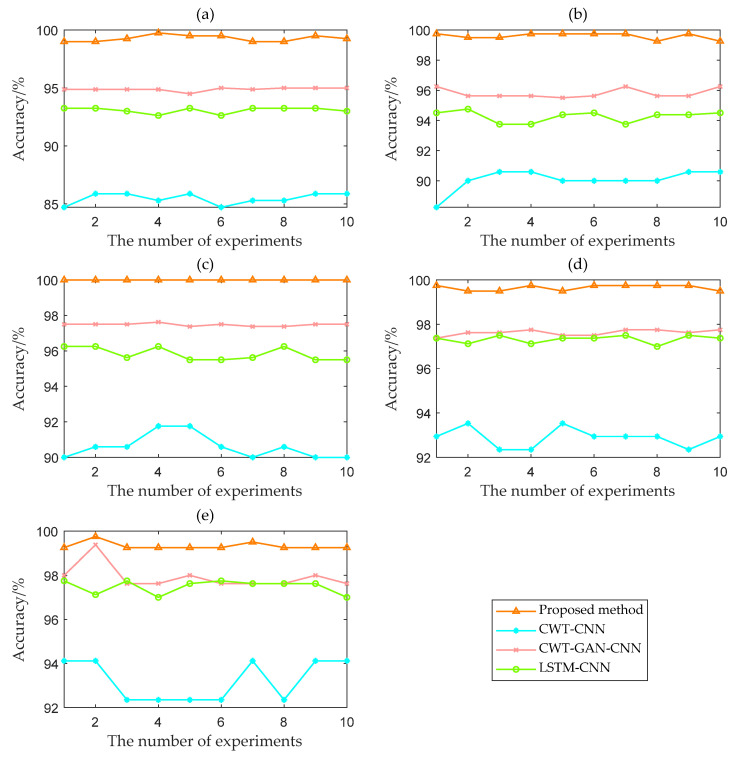
Accuracy curves of four models with different SNRs: (**a**) SNR = −4 dB; (**b**) SNR = −2 dB; (**c**) SNR = 0 dB; (**d**) SNR = 2 dB; (**e**) SNR = 4 dB.

**Figure 14:**
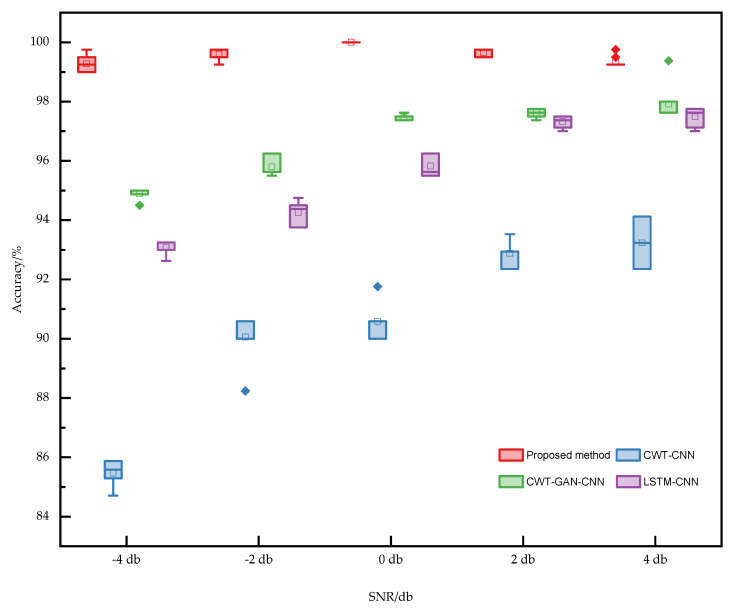
Comparison of the performance of different models with different SNRs. It mainly contains four models, including the proposed method, CWT−CNN, CWT−GAN−CNN, and LSTM−CNN.

Figure 13 shows the variation of the 10-test accuracy for the four methods at five SNRs. Figure 14 shows the box plot based on the accuracy of ten times. The proposed TFFO and CNN-based imbalance fault diagnosis approach have more than 99% accuracy at different SNRs. In contrast, the test accuracies of model CWT-GAN-CNN and model LSTM-CNN in a −4 dB noise environment are only about 95% and 93%, respectively. The diagnostic performance of the GAN and LSTM networks is approximately the same at each SNR but slightly lower than the TFFO. CWT-CNN method has the most significant variance in accuracy values at each SNR, and the model is the most unstable. It is difficult for CWT-CNN models to identify fault types when the data set is severely imbalanced. In a word, the TFFO-CNN approach shows optimal performance in terms of accuracy and stability.

For example, the data length of 12,000 points is used to expand the sample, the proposed TFFO method takes about 5 min, the GAN model takes 100 min, and the LSTM model takes 70 min. This is because the TFFO data augmentation method generates new samples by oversampling the time-frequency features. GAN and LSTM, on the other hand, require continuous training and refinement of the minority sample. Thereby, the approach proposed is also much better than other data-enhancement methods in terms of timeliness.

The hyperparameters in the proposed CNN are the optimal values of multiple artificial experiments. To further explore the effect of hyperparameters on the classification results, we perform experimental analyses on different combinations of three parameters of batch size, learning rate, and dropout using Dataset 3. As we can see from Experiment 1 in Table 4, the diagnostic accuracy reaches 100% when the batch size is over 50. However, when the batch size is too large, the model requires more epochs for training and a higher RAM. Therefore, the model is optimal when the batch size is 50. Meanwhile, when the learning rate is 0.001, the result is optimal from Experiment 2. We can see that the value of dropout has no effect on the diagnostic results using Dataset 3 from Experiment 3. However, the model is prone to overfitting when the amount of data is small. In fact, the dropout technique can effectively solve the model overfitting phenomenon.

Further, experiments are conducted on Dataset 1, which has smaller data, and the diagnostic results are shown in Table 5. Different dropout values have a significant impact on the diagnostic accuracy of the model, and the result reaches the optimum when the dropout is 0.5. Generally, the value of dropout is set to 0.5, which is a reasonable approximation to taking the geometric mean of the predictive distributions produced by the exponentially-many dropout networks [53].

In summary, when the model is optimal, the batch size, learning rate, and dropout are 50, 0.001, and 0.5 in this paper, respectively.

### 4.2. Case Study 2: The Gearbox Dataset

#### 4.2.1. Experimental Setup

**Figure 15:**
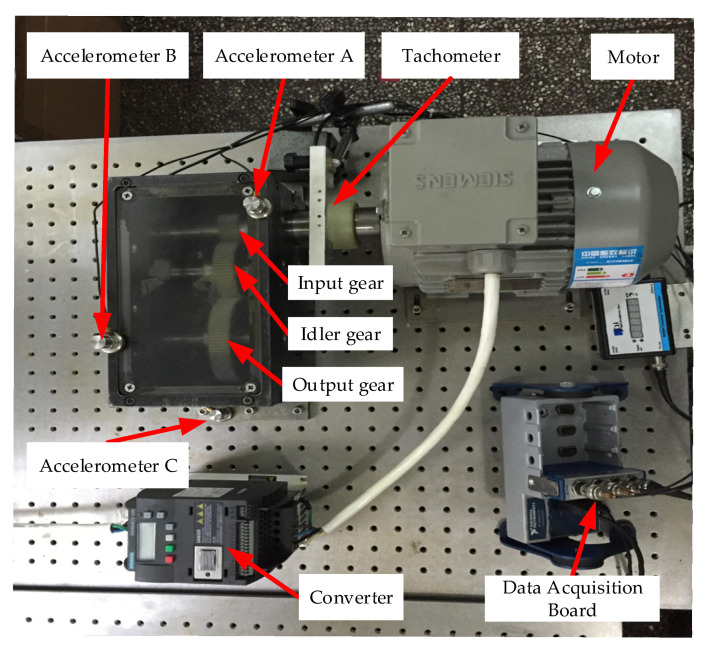
The gear test rig, which is from Zhejiang University and primarily contains a motor, three gears, and three accelerometers, and a data acquisition board.

In this experiment, the gearbox dataset from Zhejiang University is used [61], and Figure 15 shows the experimental gear rig, which comprises an AC motor, coupling, and a two-stage gearbox. The driving power of the motor is 0.75 kW, and the maximum speed can reach 3000 r/min. The frequency converter controls the speed of the vehicle. The experiment uses three single-axis accelerometers fixed at the gearbox’s input, output, and mounting plates to collect vibration signals at different locations. The number of teeth of the input, inert, and output gears is 32, 64, and 96, respectively. However, the gear may have a missing tooth, broken teeth, a crack in the tooth root, and gluing and peeling of the tooth surface. Table 4 provides a detailed description of the ten health conditions. The sampling frequency is 25.6 kHz, and the rotating speed is 2700 rpm during the experiment.

#### 4.2.2. Experimental Results 

For the 10-classified gear data set, there are 240,000 data points for health status and 50,400 for each of the other nine types of fault status, and the proportion of class imbalance is about 4.76. The rotation of the gear with the maximum number of teeth according to the sampling frequency and rotational speed will produce about 569 points in one cycle. The sample length of this paper is 1200. Table 6 shows the sample changes before and after secondary data expansion.

This article adopts four performance indicators, accuracy, precision, recall and F1-score to indicate diagnosis ability with test data, as shown in Table 7. A higher value means better fault diagnosis performance. The CWT-CNN method is applied as a comparison method using an unbalanced dataset, while the remaining two methods use different sample expansions. Compared to the other three methods, the method proposed in this paper improved accuracy by 18.35%, 2.47%, and 7.19%, respectively. The precision increased by 19.72%, 2.39%, and 7.17%, respectively. The recall rate increased by 17.48%, 2.67%, and 6.73%, respectively. The improvement in F1-score is 18.83%, 2.53% and 6.77%, respectively. In the comparative analysis of the above data, it can be seen that the proposed approach outperforms the other three methods in all metrics, which indicates that TFFO-CNN has excellent diagnostic performance.

### 4.3. Discussion

This paper proposes an imbalanced fault diagnosis method based on time-frequency feature oversampling and CNN for rotating machinery. First, this paper adopts the first expansion of the fault data from the sliding segmentation method. Subsequently, the sample performs feature enhancement and denoising by the TFFO method. Finally, CNN completes the fault identification of the balanced dataset. In the analysis, three imbalanced scale datasets are constructed to verify the diagnostic performance of the model. The bearing data set is the actual operational failures of the wheelset bearings. It is challenging for researchers to obtain the fault data, but they are significant for applying diagnostic models under realistic operating conditions. Meanwhile, the robustness of the model is examined under different SNRs. The experiments were compared with three methods, CWT-CNN, CWT-GAN-CNN, and LSTM-CNN. Ten times diagnostic accuracy and box plot results show that the proposed approach outperforms the other methods in accuracy and stability in all cases. The proposed approach takes less time to obtain higher diagnostic accuracy when processing image data. The reason is that the TFFO method is a feature-based oversampling method that is more time sensitive. Four comprehensive evaluation metrics of the laboratory artificially faulty gear dataset were extracted, indicating that the proposed method still has a high fault identification capability when dealing with other diagnostic objects and imbalanced ratios. In fact, the data expansion method proposed in this paper is not limited to the imbalance ratio in the text. It can be applied to other fault diagnosis tasks with imbalanced data sets.

## 5. Conclusions

This paper focuses on the imbalanced fault diagnosis problem and proposes a TFFO-CNN-based model characterized by the development of a time-frequency feature oversampling technique to reconstruct robust class balance data and further feature extraction and fault classification using the 2dCNN model. This combination gives full play to the advantages of each model. The main conclusions are summarized as follows:

(1) The proposed model constructs balanced datasets by simultaneously extending the time-domain signal and time-frequency domain features, which performs a comprehensive data expansion from different dimensions.
(2) Applying the CWT to convert vibration signals into image data allows the signal to achieve denoising and automatic feature extraction. SMOTE oversampling method is performed on the denoised time-frequency features to generate high-quality samples, which solves the problem that the other sample expansion methods do not consider the noise and result in the low quality of the generated data, such as GAN and LSTM. The time-frequency feature oversampling method that combined CWT and SMOTE can significantly reduce the sample generation time.
(3) The proposed imbalance fault diagnosis model solves the problem of inadequate model training effectively under a variety of imbalanced radios. The proposed imbalance fault diagnosis approach has more than 99% accuracy at different SNRs using bearing dataset 3. Meanwhile, compared to the other methods, including CWT-CNN, CWT-GAN-CNN, and LSTM-CNN, the method proposed in this paper improved accuracy by 18.35%, 2.47%, and 7.19% in the gear dataset, respectively. Experiments prove that the final fault recognition rate of the imbalance fault diagnosis model of rotating machinery based on TFFO, and CNN is the best among the models tested.

This approach provides a solution for imbalanced fault diagnosis of rotating machinery and demonstrates the potential of combining the time-frequency feature oversampling technique with the CNN model in fault diagnosis. While good results have been obtained using the proposed method based on bearing and gearbox datasets, further discussion is still necessary on the failure of rotating machinery where interrupted shaft failures and rotor failures, etc., also often occur. We will evaluate the proposed method with the rotor datasets in future work. Moreover, the study will further examine the performance of the proposed method for the case of compound fault diagnosis in the actual industry.

## Figures and Tables

**Table 1:** A detailed description of the bearing data set.

Label	Fault Type	Length	Original Samples	Dataset 1	Dataset 2	Dataset 3
F1	Slight failure of cage	102400	42 × 2400	50 × 2400	50 × 2400	50 × 2400
F2	Compound failure of cage and rolling body	102400	42 × 2400	50 × 2400	50 × 2400	50 × 2400
F3	Slight failure of rolling body	102400	42 × 2400	50 × 2400	50 × 2400	50 × 2400
F4	Slight failure of inner ring	102400	42 × 2400	50 × 2400	50 × 2400	50 × 2400
F5	Severe failure of inner ring	102400	42 × 2400	50 × 2400	50 × 2400	50 × 2400
F6	Slight failure of outer ring	102400	42 × 2400	50 × 2400	50 × 2400	50 × 2400
F7	Severe failure of outer ring	102400	42 × 2400	50 × 2400	50 × 2400	50 × 2400
F8	Normal	1200000	500 × 2400	50 × 2400	250 × 2400	500 × 2400

**Table 2:** The detailed structure of CNN.

Layer	Kernel	Strides	Output Size	Activation	Padding	Param
Input	/	/	98 × 2400 × 1	/	/	0
C1	4 × 4	4	24 × 600 × 64	ReLU	Valid	1088
S1	2 × 2	2	12 × 300 × 64	/	/	0
C2	2 × 2	2	6 × 150 × 128	ReLU	Valid	32,896
S2	2 × 2	2	3 × 75 × 128	/	/	0
F1	128	/	128	Sigmoid	/	3,686,528
F2	N	/	N	Softmax	/	1032

**Table 3:** Comparison of the performance comparison of different SNRs.

Dataset	Judging Criteria/%	−4 dB	−2 dB	0 dB	2 dB	4 dB
Dataset 1	Average accuracy	91.38	93.625	98.75	93	95.5
	Max-Min	6.25	8.75	2.5	2.5	2.5
Dataset 2	Average accuracy	97.75	97.15	99.35	98.3	98.8
	Max-Min	0.5	1.25	1	1.25	0.75
Dataset 3	Average accuracy	99.275	99.6	100	99.65	99.325
	Max-Min	0.75	0.5	0	0.25	0.5

**Table 4:** Experiments for selection of optimal parameters using Dataset3.

Experiments	Initial Conditions	Variants	Test 1	Test 2	Test 3	Test 4	Test 5
1	Learning rate = 0.01 Dropout = 0.5	Batch size	30	40	50	60	70
		Accuracy	98.4%	99.1%	100%	100%	100%
2	Batch size = 50 Dropout = 0.5	Learning rate	0.0001	0.001	0.01	0.1	1
		Accuracy	99.2%	100%	97.9%	13.4%	12.5%
3	Batch size = 50 Learning rate = 0.01	Dropout	0	0.3	0.5	0.7	0.9
		Accuracy	100%	100%	100%	100%	100%

**Table 5:** Experiments for selection of optimal dropout using Dataset 1.

Initial Conditions	Variants	Test 1	Test 2	Test 3	Test 4	Test 5
Batch size = 50 Learning rate = 0.01	Dropout	0	0.3	0.5	0.7	0.9
	Accuracy	97.2%	98.67%	100%	97.9%	69.4%

**Table 6:** Introduction to gear data sets.

Label	Fault Type and Condition	Samples	Second Enhancement
C1	a broken tooth on the input gear	42 × 1200	200 × 1200
C2	a pitted tooth on the input gear	42 × 1200	200 × 1200
C3	a pitted tooth on the idler gear	42 × 1200	200 × 1200
C4	a pitted tooth and broken tooth on the output gear	42 × 1200	200 × 1200
C5	a missing tooth on the output gear	42 × 1200	200 × 1200
C6	a cracked tooth on the input gear	42 × 1200	200 × 1200
C7	a cracked tooth on the idler gear	42 × 1200	200 × 1200
C8	a cracked tooth on the output gear	42 × 1200	200 × 1200
C9	a broken tooth on the input gear and a pitted tooth on the idler gear	42 × 1200	200 × 1200
C10	normal	200 × 1200	/

**Table 7:** Evaluation indicators for different models.

Criteria/%	Proposed Method	CWT-CNN	CWT-GAN-CNN	LSTM-CNN
Accuracy	99.50 ± 0.25	81.15 ± 1.54	97.03 ± 1.16	92.31 ± 1.54
Precision	99.25 ± 0.50	79.53 ± 0.89	96.86 ± 0.24	92.08 ± 0.78
Recall	98.71 ± 0.30	81.23 ± 0.93	96.04 ± 1.03	91.98 ± 0.34
F1-score	98.79 ± 0.29	79.96 ± 1.08	96.26 ± 0.51	92.02 ± 0.33

## Data Availability

Data are available from the authors upon request.

## Abbreviations

TFFO	Time-Frequency Feature Oversampling Technique
CNN	Convolution Neural Networks
CWT	Continuous Wavelet Transform
GAN	Generating Adversarial Networks
RNN	Recurrent Neural Networks
VAE	Variational Auto-Encoder
SMOTE	Synthetic Minority Oversampling Technique
SVM	Support Vector Machine
WT	Wavelet Transform
SNR	Signal-to-Noise Ratios
LSTM	Long Short-Term Memory Network

## Mathematical Notations

$M=\frac{N-W+B}{B}$	*M* is the number of samples after sliding segmentation *N* is the sample length *W* is the slip window size *B* is the moving step size
$X_{new}=X_{0}+w(X-X_{0})$	$X_{new}$ is the generated point $X_{0}$ is the minority category X is the surrounding sample w is the uniform random variable in the range (0,1)
$x(t)\in L^{2}(R)$	x(t) is the vibration signal $L^{2}(R)$ is the Hilbert Space
$w_{wt}(a,b)=\frac{1}{\sqrt{a}}\int_{-\infty }^{+\infty }x(t)\;\psi_{a,b}(\frac{t-b}{a})dt$	a is the translation factor b is the scale parameter $\psi_{a,b}$ is a family of wavelet functions $w_{wt}(a,b)$ is the wavelet transform
